# Detained Persons Incarcerated for the First Time and Needing Acute Psychiatric Care: Sociodemographic and Clinical Characteristics

**DOI:** 10.3389/fpsyt.2022.904735

**Published:** 2022-06-28

**Authors:** Isabella D'Orta, Nicolas Guilbert, Mathieu Pierrard, François R. Herrmann, Panteleimon Giannakopoulos

**Affiliations:** ^1^Division of Institutional Measures, Medical Direction, Geneva University Hospitals, Geneva, Switzerland; ^2^Institute of Global Health, University of Geneva, Geneva, Switzerland; ^3^Department of Rehabilitation and Geriatrics, Geneva University Hospitals and University of Geneva, Geneva, Switzerland; ^4^Department of Psychiatry, University of Geneva, Geneva, Switzerland

**Keywords:** acute psychiatric care, depression, first time inmates, prison, psychiatry

## Abstract

Among detained persons, those incarcerated for the first time (FTI: first time incarceration) are known to present long-standing psychological vulnerability but also suffer significant deterioration of their mental health during the first year following imprisonment. Whether the patterns of psychiatric morbidity differ in FTI cases compared to cases with repeated and long term incarceration (RLTI) is still a matter of debate. We examined the sociodemographic and clinical differences between a subgroup of FTI vs. one of RLTI in a series of 139 randomly selected detained persons admitted to an acute psychiatric ward located in the central prison of Geneva, Switzerland. Fisher exact, unpaired Student t and Mann-Whitney U tests were used to explore sociodemographic (age, gender, marital status, religion, knowledge of French, education) and clinical (psychiatric outpatient care, suicidal behavior, psychiatric diagnosis) differences between the two groups. Subsequently, univariate and multiple logistic regression models were used to detect the variables associated with FTI. The proportion of women was significantly higher in the FTI compared to the RLTI group. FTI cases were also more frequently separated or divorced, with less frequent religious affiliation. 16.9% of FTI cases but only 1.3% of RLTI cases had a clinical diagnosis of depression. In multiple regression models, female sex and lower religious affiliation rate were associated with FTI status. Among diagnostic categories, depression was strongly related to FTI status both in univariate and multivariable models. Importantly, this was not the case for adjustment disorders, previous history of psychiatric care and suicidal behavior. Our observations support the assumption that FTI cases with lower affective support, less religious investment and without psychiatric care prior to imprisonment are particularly vulnerable to depressive illness.

## Introduction

Admission to prison is known to be a highly sensitive period for those sentenced to this punishment. As already highlighted by Reinhardt and Rogers (1), the imprisonment may be a stressful event especially during its first weeks. Unlike long-term detained persons, newly incarcerated persons need to adapt to an environment with which they lack previous experience (2). In a very short lapse of time, they need to cope with confinement and pervasive deprivation (loss of liberty, of autonomy, of goods and services) (3). Dealing with social disapproval, feelings of guilt and shame, separation from family and friends and violence among detained persons may be additional stressors for this group (4, 5). During the first weeks of detention, there is an exacerbation of anxiety and feelings of hopelessness, loneliness and loss (3).

Longitudinal studies on the course of mental health issues are still scarce and with contradictory results. First time incarceration (FTI) is associated with long-standing psychological vulnerability and significant deterioration of mental health during the first year following imprisonment (6, 7). Moreover, the shortage of mental health services in prisons may lead to care interventions that focus on serious conditions (such as acute psychotic episodes) neglecting the symptoms of depressive and anxious disorders (3). However, a systematic review of 15 studies (8) suggests a long-term improvement of mental health symptoms during incarceration, especially those related to depression and anxiety (9). Increased access to health care, reduced consumption of alcohol and drugs, and fulfillment of basic needs may partly explain this phenomenon. Among chronically evolving pathologies, borderline personality disorder was diagnosed in more than 20% of women and 10% of men admitted to prison for the first time. Furthermore, 17–30% of men and 10–24% of women suffer from alcohol misuse or dependence while 10–48% of men and 30–60% of women were diagnosed with an illegal drug pathology upon admission to prison (10). In addition, almost 50% of FTI cases satisfy the criteria for an anxiety disorder, and more than 30% for an affective disorder (11, 12). Self-harm and past psychiatric diagnosis were the main determinants of their admission to mental health services (13, 14).

Compared to detained persons with repeated and long-term incarceration (RLTI), FTI cases are thought to be less socially disadvantaged, with lower exposure to drug addiction (15). They show a higher sensitivity to anxiety with higher rates of generalized anxiety disorders and panic attacks (16, 17). However, other studies presented conflicting data regarding the differences between these two groups challenging the idea of a particular effect of first imprisonment on the psychological well-being of detained persons (18–21). To get better insight into this issue, the present study investigates FTI and RLTI cases with psychiatric disorders admitted to an acute care ward located in the central prison of Geneva, Switzerland. In particular, we aimed to explore whether these groups differ in terms of sociodemographic (age, gender, marital status, religion, French knowledge, education) and clinical variables (psychiatric outpatient care, suicidal behavior, psychiatric diagnosis).

## Materials and Methods

### Subjects

We examined the psychiatric records corresponding to all admissions during a 5-year period (2014–2019) in UHPP (Unité hospitalière de psychiatrie pénitentiaire), a unit of 15 beds specially designed for acute psychiatric care of detained persons from the French speaking counties. This ward is located in a medium-security hospital. Two groups of patients may be admitted to this ward: regular prison detained persons and offenders with court-ordered treatments because of chronic mental disorders and high risk of recidivism. Admission to the UHPP was based on the presence of acute symptoms associated with self or others-threatening behavior and need for urgent psychiatric care. The total number of admissions for the period of reference was of 1305. The mean length of stay was of 20.7 days.

### Procedure

Multiple admissions were registered in 71.3% of cases (*n* = 930) whereas the remaining 28.7% of cases (*n*= 375) were admitted only once during the period of reference. To prevent overweighting of those repeatedly admitted, we randomly selected 100 cases for each group (repeated and single admissions). In order to avoid biases related to the long-standing psychiatric vulnerability of detained persons with court-ordered treatments (*n* = 61 among the 200 randomly selected cases), we excluded this population from further statistical analysis.

The final sample was formed by 139 detained persons (mean age: 31.7 ± 10, age range: 20–42). The distinction between FTI (*n* = 59) and RLTI (*n* = 80) cases was based on the full criminal records that were available for all of these cases. These records included prior convictions in Switzerland and countries of the European Union. Convictions in other countries (including the native) were assessed on the basis of self-reports during the hospital stay. The RLTI group included detained persons with repeated convictions as well as those with a single conviction longer than 3 months during the period of reference. All of the FTI cases were incarcerated for <3 months during the period of reference.

### Measures

Each patient was assigned an identification number that was derived from the name and birth date and subsequently encrypted. Sociodemographic data included age, gender, marital status (at initial admission), education attainment (binary variable based on obligatory vs. high school education; cut-off: 9 years), most frequently spoken language (French, English or Arabic), religious group (none, Christian, Muslim, others). The psychiatric history including outpatient care and previous inpatient stays prior to incarceration was also recorded based on the clinical records. Most often spoken language instead of citizenship was used as an independent variable since this latter is influenced by dual nationality issues (Swiss citizens born in immigrant families) and did not adequately reflect the cultural background of detained persons. All of the ICD-10 clinical diagnoses were made prospectively by two independent, board-certified psychiatrists (at admission and during the hospital stay), not aware of the scope of the study. Only cases with concordant psychiatric diagnoses were considered in this sample.

### Statistical Analysis

Fisher exact, unpaired Student t and Mann-Whitney U tests were used to compare sociodemographic (age, gender, marital status, religion, French knowledge, education) and clinical variables (psychiatric outpatient care, suicidal behavior, psychiatric diagnosis) between FTI and RLTI cases. Marital status (married, separated-divorced, single), religion (none, Christian, Muslim, others) were treated as ordinal variables. French knowledge (yes/no), education (cut-off of 9 years) were treated as binary variables. This was also the case for psychiatric outpatient care and suicidal behavior. Psychiatric diagnoses included adjustment disorders (ICD 10 code F43), bipolar disorder (ICD 10 codes F30–F31), depressive disorders (ICD-10 codes F32–F33), personality disorders (ICD 10 codes F60–F61), anxiety disorders (ICD 10 code F40–F42) and psychosis (ICD-10 codes F20–F29) as well as substance use disorders (ICD-10 codes F10–F19). Cases with multiple diagnoses were considered in each diagnostic group separately. The significance level was set at P < 0.05 but was corrected to *P* < 0.00069 for multiple testing by using the Benjamini-Hochberg method (22). Univariate and multiple logistic regression models were used to assess the association between FTI (dependent variable) and sociodemographic and clinical variables. All statistical analyses were performed using Stata 16.1.

## Results

### Descriptive Data

The sample included 80 RLTI (57.6%) and 59 FTI (42.4%) cases. Among RLTI cases, 21% were incarcerated for more than 2 years during the period of reference, 62% between 6 and 24 months and 17% between 3 and 6 months. The proportion of violent crimes was similar between the two groups (69% in FTI and 71% in RLTI cases). As expected, the proportion of cases with antisocial personality was higher among RLTI compared to FTI (50% borderline, 4.5% antisocial, 45.5% other in FTI; 18.9% borderline, 20.5% antisocial, 60.6% other in RLTI). After stringent correction for multiple comparisons, there were no significant differences in these variables between the two groups.

### Group Comparisons

The proportion of women was significantly higher among FTI compared to RLTI cases. FTI cases were more frequently separated or divorced (there was only one widow; trend of significance), with a lower religious affiliation rate. In contrast, there were no significant differences in age, education level and knowledge of French between the two groups (Table 1). History of psychiatric outpatient care (before conviction), use of inpatient care (number of hospitalizations during the past 12-month period) and suicidal behavior did not differentiate FTI from RLTI cases. Regarding clinical diagnosis, depression was significantly more frequent in FTI than RLTI cases (16.9% vs. 1.3%). There were no statistically significant group differences in the distribution of the other psychiatric diagnoses (Table 1). When adjusting for multiple comparisons, the only significant difference in group comparisons concerned the higher occurrence of depression in FTI cases.

**Table 1:** Demographic and clinical differences according to FTI vs. RLTI status.

	**FTI**	**RLTI**	**Total**	***P*** **-values**
N	80 (57.6%)	59 (42.4%)	139 (100%)	
Age	30.8 ± 7.9	33.0 ± 12.2	31.7 ± 10.0	0.2137
Marital status				0.0566
Married	22 (27.5%)	10 (16.9%)	32 (23.0%)	
Separated-divorced-widowed	8 (10.0%)	14 (23.7%)	22 (15.8%)	
Single	50 (62.5%)	35 (59.3%)	85 (61.2%)	
Religion				**0.0461**
None	19 (23.8%)	27 (45.8%)	46 (33.1%)	
Christian	23 (28.7%)	14 (23.7%)	37 (26.6%)	
Muslim	37 (46.3%)	17 (28.8%)	54 (38.8%)	
Other	1 (1.3%)	1 (1.7%)	2 (1.4%)	
Male sex	74 (92.5%)	46 (78.0%)	120 (86.3%)	**0.0227**
French speaking	59 (73.8%)	42 (71.2%)	101 (72.7%)	0.8476
Education > 9 years	9 (11.3%)	13 (22.0%)	22 (15.8%)	0.1024
HighUsers_				0.4837
Mono	46 (57.5%)	38 (64.4%)	84 (60.4%)	
High	34 (42.5%)	21 (35.6%)	55 (39.6%)	
Psychiatric outpatient care	53 (66.3%)	44 (74.6%)	97 (69.8%)	0.3515
Psychiatric inpatient care	34 (43.0%)	28 (49.1%)	62 (45.6%)	0.4915
Suicidal behavior	35 (43.8%)	32 (54.2%)	67 (48.2%)	0.2345
Substance use disorders	44 (55.0%)	27 (45.8%)	71 (51.1%)	0.3068
Adjustment disorders	23 (28.7%)	15 (25.4%)	38 (27.3%)	0.7039
Bipolar disorder	4 (5.0%)	2 (3.4%)	6 (4.3%)	1.0000
Depressive disorder	1 (1.3%)	10 (16.9%)	11 (7.9%)	**0.0008**
Personality disorders	24 (30.0%)	16 (27.1%)	40 (28.8%)	0.8499
Psychotic disorders	27 (33.8%)	14 (23.7%)	41 (29.5%)	0.2592

*Bold values indicate P <0.05*.

### Regression Models

Among the variables included in group comparisons, gender (male), marital status (married), religious affiliation (Muslim), were all negatively associated with FTI status in univariate regression models. Clinically, there was a strong positive association between FTI status and the diagnosis of depression. To consider the interdependence of some independent variables, multivariable models were also considered. In multivariable models, lower religious affiliation rate (Christian and Muslim) and diagnosis of depression were both significantly related to FTI (Table 2).

**Table 2:** Univariate and multiple logistic regression analysis of factors associated with FTI status.

	**Univariate**		**Multiple**	
	**OR (95% CI)**	***P*** **-values**	**OR (95% CI)**	***P*** **-values**
Age	1.022 (0.987,1.057)	0.217	1.012 (0.970,1.056)	0.581
Male sex	0.287 (0.102,0.808)	**0.018**	0.300 (0.094,0.956)	**0.042**
**Marital status**				
Married	1.000 (1.000,1.000)		1.000 (1.000,1.000)	
Separated-divorced-widowed	3.850 (1.224,12.111)	**0.021**	3.229 (0.884,11.792)	0.076
Single	1.540 (0.650,3.651)	0.327	2.367 (0.852,6.574)	0.098
**Religion**				
None	1.000 (1.000,1.000)		1.000 (1.000,1.000)	
Christian	0.428 (0.177,1.039)	0.061	0.355 (0.132,0.954)	**0.040**
Muslim	0.323 (0.142,0.735)	**0.007**	0.332 (0.131,0.842)	**0.020**
Other	0.704 (0.041,11.964)	0.808	0.908 (0.045,18.116)	0.949
	0.690 (0.351,1.357)	0.282	1.053 (0.469,2.364)	0.901
Depressive disorder	16.122 (2.002,129.867)	**0.009**	20.056 (2.244,179.258)	**0.007**

*Bold values correspond to the title of nominal items*.

## Discussion

In the present study, the occurrence of depression was significantly higher in FTI compared to RLTI cases after controlling for sociodemographic factors and other psychiatric morbidities. This strong association persisted even after the correction for multiple comparisons. Importantly, the probability of depressive illness was more than 20 times higher in FTI compared to RLTI cases needing acute psychiatric care. This finding is consistent with previous studies reporting that severe depression is a prevalent mental health condition among novice prison populations (2). Other studies also show that FTI is associated with severe difficulties in adjusting to prison reality (23) that can rapidly lead to clinically overt depression (2). Unlike depression, there was no group difference in the occurrence of adjustment disorders in the present series implying that the depressive symptoms in our FTI cohort is not a simple consequence of the difficulties related to loss of freedom and confrontation with the penitentiary system. Also, similar frequency of suicidal behavior among FTI and RLTI cases indicates that the acute distress related to first imprisonment does not favor, in our sample, self-threatening attitudes. The discrepancy observed between formal diagnosis of depression and suicidal behavior is due to the high proportion of cases that display self-threatening attitudes during their incarceration without satisfying the ICD-10 criteria of depressive illness. In fact, only 7.9% of the whole group met the criteria for depression with a clear over-representation among FTI cases (16.9% of FTI but only 1.3% of RLTI cases display clinically overt depression). Psychiatric outpatient or inpatient care was present in most FTI cases but this was also the case for RLTI cases without significant differences between the two groups.

Besides this main observation, some additional findings need to be considered. Our FTI cases include a larger proportion of women, separated or divorced individuals with lower religious affiliation rate. The population of women in prison worldwide is drastically increasing (at a faster pace than men's) with evidence of high rates of psychiatric morbidities (24, 25). There is an international consensus that women in prison are particularly vulnerable (26). The present results suggest that compared to men, women are more prone to develop acute psychiatric symptoms requiring hospitalization in the initial period of their incarceration. This may be partly explained by the significant distress experienced by women entering prison for the first time. Penitentiaries are built and organized for men and all procedures, including mental health care treatments and follow-ups do not usually take into account gender specificities. The need for rapid psychiatric hospitalization after conviction may not only reflect an already existing mental health burden, but possibly also the overall adversity for women facing the penitentiary system. Hospitalized FTI cases were also more frequently separated or divorced and showed lower religious affiliation rates than RLTI cases. These results support previous findings which emphasize the importance of social and affective relationships to maintain mental well-being and promote adjustment in prison life for FTI (27). These data imply that the absence of such support is much more critical during the first months of incarceration than for recidivists or detained persons with long term convictions. Among these variables, lower religion affiliation rate and female sex remained significant predictors of the FTI status in multivariable models. These two socio-demographic parameters may thus represent risk factors that in combination with depressive symptoms steadily increase the risk of psychiatric hospitalization among FTI cases.

From a clinical viewpoint, the increased vulnerability of FTI cases to depressive illness should be taken into account in the definition of mental health strategies that favor its early detection and treatment in this subgroup of detained persons. Strengths of the present study include the admission of all cases in the same unit of acute psychiatric care in prison that decreases the variability in the admission criteria, and use of multivariable models that allow for controlling the interdependence between the clinical and demographic variables. To avoid biases related to the long-standing psychiatric vulnerability of detained persons with court-ordered treatments, we did not consider this subgroup in our analysis. Several limitations should, however, be taken into account. To be close to a real-life situation, clinical diagnosis was carried out using two independent clinicians unaware of the scope of the study but without use of standardized diagnostic questionnaires. Binary data on religious practice and spoken language may mask more complex realities with respect to the ethnic and cultural background of the detained persons in both groups. Two main selection biases were also identified using the STrengthening the Reporting of OBservational studies in Epidemiology (STROBE) protocol (28). First, the vast majority of RLTI cases had an incarceration duration that exceeded 6 months, yet in 17% of cases detained persons were repeatedly incarcerated for short time periods (<6 months during the period of reference). Moreover, criminal records included prior convictions in Switzerland and countries of the European Union. Convictions in other countries (including the native) were assessed on the basis of self-reports during the hospital stay. We cannot thus exclude a declaration bias that could affect the composition of the RLTI group. Similarly, the difference in time spent in prison may impact *per se* on the psychological vulnerability of RLTI (with a possible improvement over time). To address partly this limitation, our randomization process prevents the overweighting of cases with repeated admissions during the period of reference. To obtain meaningful differences with well-established diagnoses, we focused on detained persons who have been referred for inpatient psychiatric care in UHPP. In regular prisons, detained persons may benefit from outpatient psychiatric consultations and display milder patterns of psychopathology (both for FTI and RLTI). In order to save statistical power, no separate analysis by subtype of personality disorders and length of incarceration was performed. Last but not least, some associations should be carefully interpreted since they did not persist when using stringent correction for multiple comparisons. Future studies in larger samples including comparisons with court-ordered treatments using standardized assessment of clinical diagnosis and demographic factors, and distinction between personality subtypes are warranted to explore the differences between FTI and RLTI cases across the different legal and psychiatric care systems in Europe.

## Data Availability Statement

The raw data supporting the conclusions of this article will be made available by the authors, without undue reservation.

## Ethics Statement

The studies involving human participants were reviewed and approved by Geneva Ethics Committee. Written informed consent for participation was not required for this study in accordance with the national legislation and the institutional requirements.

## Author Contributions

ID'O and PG contributed to the conception, design of the study, and wrote the paper. ID'O, NG, and MP were involved in the acquisition of data. ID'O supervised the database. FH performed the statistical analysis. All authors contributed to manuscript revision and approved the submitted version.

## Funding

Open access funding was provided by the University of Geneva.

## Conflict of Interest

The authors declare that the research was conducted in the absence of any commercial or financial relationships that could be construed as a potential conflict of interest.

## Publisher's Note

All claims expressed in this article are solely those of the authors and do not necessarily represent those of their affiliated organizations, or those of the publisher, the editors and the reviewers. Any product that may be evaluated in this article, or claim that may be made by its manufacturer, is not guaranteed or endorsed by the publisher.
