# Erratum: Detained persons incarcerated for the first time and needing acute psychiatric care: Sociodemographic and clinical characteristics

**DOI:** 10.3389/fpsyt.2023.1182157

**Published:** 2023-03-21

**Authors:** Frontiers Production Office

**Affiliations:** Frontiers Media SA, Lausanne, Switzerland

**Keywords:** acute psychiatric care, depression, first time inmates, prison, psychiatry

An omission to the funding section of the original article was made in error. The following sentence has been added: “Open access funding was provided by the University of Geneva.”

The original article has been updated.

